# History of Medical Teaching in Texas

**Published:** 1906-11

**Authors:** J. F. Y. Paine

**Affiliations:** Professor of Obstetrics, Medical Department University of Texas, Galveston


### TEXAS MEDICAL JOURNAL.

Established July, 1885.

F. E. DANIEL, M. D., - - - - Editor, Publisher and Proprietor.

PUBLISHED MONTHLY.—SUBSCRIPTION $1.00 A YEAR.

VOL. XXII. AUSTIN, NOVEMBER, 1906. No. 5.

The publisher is not responsible for the views of contributors.

For Texas Medical Journal.

### History of Medical Teaching in Texas.

BY J. F. Y. PAINE, M. D., PROFESSOR OF OBSTETRICS, MEDICAL DEPARTMENT UNIVERSITY OF TEXAS, GALVESTON.

At the opening of the Sixteenth Annual Session of the Texas University Medical College at Galveston, October, 1906, Professor Paine delivered the following address:

*Mr. President, Ladies and Gentlemen, and Ladies and Gentlemen of the Classes in Medicine, Pharmacy and Nursing:*

It is my pleasure on behalf of the faculty to extend to you students of the respective classes, who have been with us before, a cordial greeting, and to you who are here for the first time, a hearty welcome.

A brief review of the medical schools which have existed in Galveston at different times has suggested itself to me as a subject that might interest you on this occasion. The first medical college in Texas was organized in Galveston in 1860, as the Medical Department of Soule University, under the name of the Galveston Medical College. This institution possessed a charter, whose provisions authorized the teaching of medicine in all of its branches, the graduation of students and conferring the degree of M. D.

The inquiry naturally arises, What circumstances could have inspired the attempt to establish a medical school at that time when conditions for the accomplishment of such an enterprise were so unpromising? The explanation lies in the fact that Texas was very sparsely inhabited, settlers living at great distances apart. Galveston, the largest town within her borders, had a population in 1860

of 7307, while the entire State in the same year contained but 421,649 souls.

It was not unusual in those days for the settler to haul his grain in an ox wagon 200 miles to a grist mill, and doctors were as infrequent as other public utilities. As a consequence of this situation of affairs, medical practice was largely in the hands of men who had acquired some experience in giving medicines to slaves, under the direction of physicians, on plantations in the older States. Every settlement claimed among its citizenship such a person, to whom the people referred their ills as well as their injuries, and whose advice and ministrations were accepted with a gratitude and confidence that would be entirely gratifying to a present-day physician. In time these men came to be called doctor. In many instances they were greatly revered, being regarded as natural-born physicians. But the medical oracles of that day were not limited to members of the male profession. I dare say an easy hunt would find even now women and men who retain unhappy recollections of perusolemoak confections and sassafras teas administered in their kid days, under helpless protest, by some old woman. The helpless condition of the widely-scattered people when suffering from medical surgical disorders or injuries was fully appreciated, and a movement was inaugurated among the citizens in the more populous sections to afford them relief. Accordingly a convention of representative physicians from different sections of the State was held in Galveston and a committee was selected to confer with the board of trustees of Soule University with the view of organizing a medical school. This conference was successful, and the Medical Department of Soule University, under the name of the Galveston Medical College, was organized in Galveston in 1860.

Soule University was founded in 1856, four years before the establishment of its medical branch, under the control and supervision of the Texas Conference of the Methodist Episcopal Church, South, and was located at Chappell Hill, in Washington county. A noteworthy feature of this institution is the prominence of the men who constituted the first board of trustees, viz.: Thomas B. White, J. D. Giddings, J. H. Davidson, J. W. Hipple, Richmond Crawford, James McLeod, Robert Alexander, H. Yoakum, Gabriel Felder, C. P. Barten, W. S. Day, H. S. Thrall, L. D. Bragg, William Chappell, J. C. Wilson, and William G. Webb.

When the war between the States had ended the people applied themselves energetically to the laborious undertaking of repairing their wasted homes and retrieving their useful institutions. Among the acts of rehabilitation, the Galveston Medical College was reorganized in 1866, under the auspices of Soule University, whose board of trustees appointed for it a corps of teachers, the first faculty of a Texas medical school of which there is any authentic record. The personnel of this faculty is as follows: Greensville Dowell, M. D., professor of anatomy; N. N. Allen, A. M., M. D., professor of surgery; W. A. East, M. D., professor of practice of medcine; John H. Webb, M. D., professor of materia medica and therapeutics; W. H. Grant, M. D., professor of physiology and pathology; William H. Boring, M. D., professor of obstetrics and diseases of women and children; De Port Smythe, M. D., professor of chemistry; Robert Hanno, M. D., demonstrator of anatomy; Greensville Dowell, M. D., dean of the faculty.

Changes in the faculty were of such common occurrence that between the date of the first reorganization in 1866 and that of the second in 1873, that a considerable number of prominent medical men of the State had occupied professors' chairs in the school. During this period the professorship of medicine was filled successively by Drs. W. D. Kelley, S. M. Welch, and T. J. Heard; of obstetrics, by Drs. R. A. Watkins, J. M. Calloway, and —. Jennings; of chemistry, by Drs. W. B. Briggs, J. A. Allen, and J. H. Webb. Dr. F. E. Daniel, of Austin, editor of the TEXAS MEDICAL JOURNAL, was the incumbent of the chair of anatomy in 1867-68. At an early day Dr. Dowell succeeded to the chair of surgery, and the mutations in the faculty did not affect his tenure of that position.

The scheme of instruction consisted of didactic lectures on anatomy, physiology, chemistry, materia medica, and therapeutics, practice of medicine, surgery, and obstetrics, dissections, hospital clinics and post-mortem examinations. The sessions continued four months. As an essential condition to graduation attendance upon two courses of lectures (five years of general practice being accepted as equivalent to one course), a thesis on some medical topic and a satisfactory examination on the several branches of medical science were required. These exercises continued annually until 1873, the date of the second organization.

The Galveston Medical College seems to have had somewhat of a nomadic experience. Its first home was in the second story of a frame building at the intersection of Postoffice and Twenty-second streets, over what was then known as Mrs. Goeppenger's restaurant. It has never been revealed whether the removal from this place was voluntary or suggested by neighbors who were not entirely in sympathy with all of the appointments of such an institution. At any rate, its next domicile was a two-story frame structure, situated in the middle of the block, on the south side of Avenue L, between Twenty-first and Twenty-second streets, where the south breezes were unobstructed. At one time this was one of the best known houses in this city, and is still spoken of by the old residents of that section as Dowell's Medical College. For many years after it was vacated by the medical school it was frequently untenanted, owing to the prevailing fear of the sight and sounds of spooks which were thought to hold nightly carnivals within its walls. And even to this day there are persons who can not pass that place at night alone without a feeling of wretched insecurity.

In the first session the equipment of the school consisted of one disarticulated skeleton, three large anatomical maps and one obstetrical manikin. Later on a few models of the eye and ear were added. It was the boast of the faculty, however, that their enthusiasm and energy compensated in large measure for the deficiency of auxiliary appliances.

The Island City Hospital was leased by Dr. Dowell in 1866, which appears to have been a part of the plan of reorganization of the medical school. Students (undergraduates) were admitted as internes and employed in various subordinate capacities in this institution. The hospital contained about 150 beds, a large proportion of which, it is said, were generally occupied, and the favorable circumstances of its management permitted the unrestrained use of patients for medical, surgical and other clinics. Internes enjoyed unlimited authority to prescribe for patients.

Material for dissection was obtained indirectly from this source. But the rigid exactions of the law had to be complied with, and to evade its penalties the dead bodies generally underwent the formality of interment. These subjects were subsequently resurrected by the students. The ghoulish forays being undertaken after midnight on the dark of the moon, the hair-raising experiences were sometimes associated with these grewsome missions.

As Galveston grew, increased hospital accommodations became necessary. A new and more modern building, with improved furnishings, was erected and a change in the administration of the institution followed, municipal control being resumed. The Island City Hospital became the City Hospital, to which the John Sealy Hospital succeeded, all having occupied the same site.

In its first session the Galveston Medical College matriculated fifteen students, of whom six were graduated. The fees were $150 for each session, and $30 for graduation (the diploma fee). This pioneer of medical education in Texas has left behind evidences of its good work in the persons of its graduates, e. g.: Dr. Sam R. Burroughs, now a prominent physician of Buffalo, in this State, and president of the State Board of Medical Examiners, and has been president of the State Medical Association; Dr. C. H. Wilkinson, for more than thirty years a leading physician in Galveston, chief surgeon of St. Mary's Infirmary for twenty-five years, and of the Gulf, Colorado & Santa Fe Railroad for fifteen years, and is now engaged in writing, the title of his latest book being "The Tragedy of Baden"; Dr. R. H. L. Bibb, of Saltillo, Mexico, chief surgeon for many years of the Mexican National Railroad; Dr. B. F. Calhoun, of Beaumont, late president of the South Texas Medical Society; Dr. W. A. McCamley, of Wharton, and many others scattered over the State.

Some of the men connected with early medical education in this State deserve mention. Chief among them is Dr. Greensville Dowell, who was the leading spirit of the enterprise, and was in some respects far above the ordinary. His education was limited, but he possessed a sturdy intellect, and was conspicuously self-reliant. He did a considerable amount of successful surgery and enjoyed, perhaps, as much reputation as an operator as any of his professional contemporaries in this section. Original, bold and resourceful, with opportunity and training, his possible achievements in surgery might have been brilliant. He devised several surgical operations, among them one for hernia, and invented a number of surgical instruments. He established and edited the first medical periodical (the *Galveston Medical Journal*) ever published in the State (1866 to 1870). He was the author of two books on medical subjects, one on yellow fever, the other on hernia. While not included among the classics on those subjects, it is conceded that they contain many valuable truths. To him is accorded priority in directing attention to the momentous fact that yellow fever is transmitted by mosquitoes (1876) five years before Dr. Finlay enunciated his theory on that subject.

Dr. Boring was a many-sided man. Besides being a skilled physician, he was an eloquent preacher. Occupying the lecturer's rostrum on obstetrics during the week days and filling one of the methodist pulpits on Sundays. It is said of him that in a large company he was asked whether, being a physician, he ever prayer for people to get sick. Without hesitation he replied: "No; I simply ask for my daily bread and the Lord knows how it is."

The Galveston Medical College did not prosper as its promoters hoped it would. It was disappointing in that it failed to enlist the support of the physicians of the State. It was acknowledged to be insufficient in equipment to meet the demands of modern methods of medical teaching, and it was not profitable to those who bore the burden of its maintenance. Dissensions arose, and Dowell being regarded as the Jonah, was petitioned to resign, which failing, the faculty resigned in a body, leaving the professor of surgery alone in his glory. And for awhile he filled all the chairs in the school. At this juncture the Soule University was removed to Louisiana and its medical department went out of existence.

In 1873, the same year that the Galveston Medical College closed its doors, the Texas Medical College and Hospital was launched under a charter which was intended to correct the defects and supply the deficiencies of its predecessor. The incorporators of the new school were: Ashbel Smith, M. D.; D. F. Stuart, M. D.; J. D. Rankin, M. D.; A. H. Goodwin, M. D.; R. A. Watkins, M. D.; J. M. Calloway, M. D.; N. N. Allen, M. D.; R. Flewellen, M. D.; T. J. Heard, M. D.; W. S. Rogers, M. D.; Greensville Dowell, M. D.; Leonidas Hudspeth, M. D.; Thomas M. Jack, E. T. Austin, John D. Rogers, A. P. Lufkin, John Dean, and Isador Dyer.

The chief argument used by those who strenuously advocated the upbuilding of a medical school was that the diseases in Texas were *sui generis*, so modified in type and treatment by climatic conditions that they could only be studied in a rational and successful way at home. Physicians, however, learned and skillful, coming from other States, especially the East and North, were regarded with distrust, until they had lived in a community long enough "to get the run of things."

This idea was a conspicuous feature of the charter of the new school, which provided for an annual appropriation of $5000 to promote clinical teaching. To insure higher qualifications of teachers it also provided for the selection of professors by the system of concours, or competitive examinations.

Immediately after the issuance of the charter the trustees, with the Sage of Evergreen (the erudite and knightly Ashbel Smith) for president, organized for business. Their first act was to appoint a board of examiners to examine applicants for professorships. This board was constituted as follows: Ashbel Smith, M. D., examiner on surgery; Leonidas Hudspeth, M. D., examiner on practice of medicine; D. F. Stuart, M. D., examiner on anatomy; R. A. Watkins, M. D., examiner on chemistry; J. M. Calloway, M. D., examiner on physiology; W. S. Rogers, M. D., examiner on obstetrics; J. M. Haden, M. D., examiner on materia medica and therapeutics.

The board of examiners organized and adopted rules for its government. In compliance with which a card to the medical profession inviting applicants for the respective chairs to appear for examination, was published in three daily newspapers for sixty days. On the date designated the examinations were held, with the result that the following physicians were recommended to and subsequently appointed by the trustees to professorships: Greenville Dowell, M. D., professor of surgery; J. D. Rankin, M. D., professor of practice; J. M. Calloway, M. D., professor of obstetrics; G. Forgerson, M. D., professor of anatomy; J. H. Webb, M. D., professor of materia medica and therapeutics; William Penny, M. D., professor of physiology and pathology; Sam R. Burroughs, M. D., professor of chemistry.

The chairs of materia medica and therapeutics, anatomy and obstetrics becoming vacant by deaths and resignation, were filled, resectively, by H. A. West, M. D., on materia medica and therapeutics; A. W. Fly, M. D., on anatomy, and J. F. Y. Paine, M. D., on obstetrics, the new professors having passed approved examinations conformably to the requirements of the board of concours.

Although valuable additions had been made to the demonstrative apparatus, it was still sadly incomplete. The didactic and clinical instruction, however, was conceded to be a decided advance of that of the antecedent school.

Like most undertakings with limited resources and great expectations, the course of the Texas Medical College and Hospital was far from roseate, but notwithstanding her vicissitudes of fortune, the trend was never downward.

It is strange that a man who has served Texas both as Republic and State in various exalted stations of honor and responsibility, having been Surgeon General of the new Republic; Minister successively to the United States, Great Britain, France, and Spain; Secretary of State, and joint Commissioner in making the first treaty with the Comanches in 1837; who was a member of the Legislature for a number of years, and served throughout the Mexican War; who raised a regiment of Texans for the Confederate service, and commanded it valiantly; who has contributed to the world's literature valuable papers on scientific, professional and agricultural topics; who in the early epidemics of yellow fever in Galveston and Houston rendered professional services gratuitously to the sufferers; who was instrumental in the establishment of the State University and president of its board of regents until the time of his death, and when but twenty years have elapsed since he was laid to rest with Lamar, Houston, and Anson Jones, is almost forgotten. Dr. Ashbel Smith was also president of the board of trustees and examiner on surgery on the board of concours of the Texas Medical College and Hospital, and was the high priest from whom inspiration was drawn in the days of her tribulations. In his official capacity he presided on commencement occasions and delivered diplomas to the graduates in Latin with a dignity and earnestness that can never be forgotten.

In 1881, when the question of separating the medical branch from the main body of the University was being agitated and all of the more populous cities in the State were recounting their claims upon that department, Galveston entered the list and made a presentment of her superior advantages as a location for a medical school. An election was held, with the result that the medical department was to be disjoined from the main university, and Galveston selected as its location. That no obstacle might be interposed in the way of the speedy carrying out of the people's wishes, the Texas Medical College and Hospital discontinued its existence.

Seven years passed, and the State's medical school, on account of the financial status of the University, was not yet established. Strenuous efforts in its behalf before successive legislatures having failed of results, placed the matter in such indefinite and unsatisfactory form that medical men throughout the State gave expression to their impatience in personal letters and through other media, urging the expediency of reopening the school, which had suspended operations to make room for the University. The time seemed ripe for the consummation of this purpose, and the trustees determined to reorganize the Texas Medical College and Hospital. Pursuant to this end they met in Galveston in the spring of 1887, filled the vacancies on their board and went actively to work to acquire facilities and appurtenances necessary for teaching medicine according to advanced modern methods. A lease of the City Hospital buildings for ten years was secured and the wards were transformed into lecture rooms, laboratories and dissecting room. The public-spirited business men of Galveston manifested their unqualified indorsement of the undertaking by the most liberal contributions of money, aggregating many thousand dollars, for the equipment of the laboratories. The John Sealy Hospital, which was completed in the year after the college was reopened, was placed under the professional control of the faculty and its patients were utilized as clinics for advanced students.

The faculty was selected, as far as possible, with a view to individual fitness for the respective chairs, and comprised:

B. E. Hadra, M. D., professor of general and clinical surgery.

H. A. West, M. D., professor of theory and practice of medicine.

J. F. Y. Paine, M. D., professor of obstetrics and diseases of women.

A. W. Fly, M. D., professor of anatomy and clinical surgery.

H. P. Cooke, M. D., professor of physiology.

Ed. Randall, Jr., M. D., professor of materia medica, therapeutics and clinical medicine.

J. H. Wysong, M. D., professor of chemistry and toxicology.

George Dock, M. D., professor of pathology.

George P. Hall, M. D., lecturer upon diseases of the eye, ear, nose and throat.

George H. Lee, M. D., demonstrator of anatomy.

At the end of the first session George H. Lee, M. D., was elected professor of anatomy, to fill the vacancy created by the resignation of Dr. Fly, and the teaching force was increased by the selection of C. W. Trueheart, M. D., as clinical professor of gynecology, and W. J. Pettus, M. D., as lecturer on diseases of the skin, genito-urinary organs and venereal diseases. Charles C. Barrell, M. D., succeeded Dr. Lee as demonstrator of anatomy.

The curriculum and methods of teaching were in line with the progressive age of medical thought, and the course of study extended over three years.

The fees for tuition were, for matriculation, $5; general ticket for all lectures and laboratory work for first year, $100, and $150 for each of the two succeeding years. The graduation fee was $30.

There were no graduates in the first year, two in the second (both of whom had taken their first course of lectures in accredited schools), and three in the third. Undergraduates at the time of the disbandment (1881) were invariably admitted to advanced standing in other reputable medical colleges.

It can be said, to the credit of this school, as of its immediate progenitor, and to the gratification of those directly connected with them, that cheapness of tuition and easy facilities for graduation were not among the inducements offered to attract students.

Although the career of the Texas Medical College and Hospital was short, being reorganized in 1888 and dissolved in 1891, there are some memorable incidents connected with its history. It set a new pace for the medical schools of the South, being the first south of the Johns Hopkins University to extend the period of study to three years of graded courses of six months each.

The first training school for nurses in the South was established in January, 1890, by an association of public-spirited and benevolent Galveston ladies, in connection with the John Sealy Hospital, and under the instruction and training of its faculty.

Of the eight professors only one received a salary. The professor of pathology (now professor of medicine in the University of Michigan) was engaged to fill this chair with the understanding that he would be paid a stipulated sum for his services. This obligation was literally complied with, although it and the maintenance of the laboratories absorbed the entire income of the institution.

When the building for the State Medical School was completed and the Legislature had provided by appropriations for the equipment and support of the establishment, the Texas Medical College and Hospital dissolved its organization and turned over to the new school all furniture and apparatus in its possession that had been acquired, mainly by gift of Galveston citizens.

The physicians of the State were largely instrumental in determining the location of the Medical Department of the University. In 1881 when the cities competing for it were engaged in an active canvass, the State Medical Association met in Waco, and naturally this question became the engrossing topic of informal discussion. So general and intense was the interest that it was injected into a general discussion. Dr. C. H. Wilkinson introduced a resolution to this effect: "It is the sense of the Texas State Medical Association that the medical department should be segregated from the main body of the University of Texas, and located at Galveston." This resolution elicited much spirited discussion, but the logic of facts, forcibly enunciated by Drs. Wilkinson, Heard, and Ashbel Smith, was irresistible, and the debate closed with odds in favor of Galveston. It may reasonably be assumed that the popular election in September, 1881, separating the medical department from the main University and locating it at Galveston, was the result of public sentiment, dependent upon the views of physicians.

The people naturally became impatient at the delay in establishing the medical department and the city council appointed a committee of physicians (consisting of Drs. J. F. Y. Paine, John M. Haden, Henry P. Cooke, C. W. Trueheart, and H. A. West)

to prepare a memorial for submission to the Governor and Legislature, directing attention to the importance to the State of early action in this matter, and in it was incorporated the following communication from Mr. George Sealy:

GALVESTON, February 9, 1887.

*Dr. J. F. Y. Paine, Chairman, Etc.:*

DEAR SIR: As the result of my conference with you, I now state that if the present Legislature should make the necessary appropriation to organize, and with the view to permanently carry on the Medical Department of the State University at Galveston, the estate of John Sealy and his widow, Mrs. Rebecca Sealy, will contribute the sum of $50,000 for the erection of a medical hospital, to belong to the State University, and to be under the control of its regents.

No connection with the building by above parties is intended, except to furnish the money to the proper authorities on the conditions above named.

This offer will continue beyond the session of the present Legislature.

A charity, to be of the greatest benefit to the public of this city, is contemplated by the will of John Sealy. Its choice is embarrassing. A hospital as above (as far as possible with such an institution), to be open and available to the public for the highest medical skill, seems to fulfill the design. But it must be at once undertaken. Very respectfully,

GEORGE SEALY.

This was the first public intimation of Mr. Sealy's intention to build a hospital.

Failure of the Legislature to accept this munificent offer was disspiriting, but after a short period of inactivity, interest in the subject was revived by a proposition to build the hospital on condition that the Texas Medical College and Hospital be reorganized and furnish its medical and surgical staff until the State's medical school should be organized. This overture was accepted, and all the terms of the agreement were fulfilled. The hospital was completed in 1889 and donated to the city.

In 1889-90 the Legislature granted by successive appropriations, aggregating $75,000, for buildings, and the city of Galveston transferred to the State the block of ground upon which they were to be erected. The John Sealy Hospital was ceded to the University in 1890 by gift of the city of Galveston to be used in connection with clinical instruction. Subsequent appropriations for the equipment and support of the institution were made and in October, 1891, the medical department was formally opened.

The Medical Department of the University of Texas is the culmination of earnest and arduous efforts in the cause of medical education, the realization of a provision in the first Constitution of the Republic of Texas in 1836, and reiterated in the Constitution of the State in 1876. While it has reached the foremost rank among the medical schools of the South, much remains to be accomplished before it measures up to the standard of excellence contemplated by its founders, fulfills its ultimate destiny, the highest honor to the State and the greatest blessing to the people.

